# Associations between Longitudinal Patterns of Substance Use and Anxiety and Depression Symptoms among a Sample of Canadian Secondary School Students

**DOI:** 10.3390/ijerph181910468

**Published:** 2021-10-05

**Authors:** Gillian C. Williams, Karen A. Patte, Mark A. Ferro, Scott T. Leatherdale

**Affiliations:** 1 School of Public Health Sciences, University of Waterloo, Waterloo, ON N2L 3G1, Canada; mark.ferro@uwaterloo.ca (M.A.F.); sleatherdale@uwaterloo.ca (S.T.L.); 2 Faculty of Applied Health Sciences, Brock University, St. Catharines, ON L2S 3A1, Canada; kpatte@brocku.ca

**Keywords:** anxiety, depression, alcohol drinking, cannabis smoking, cigarette smoking, vaping, adolescent

## Abstract

The objective of this study is to examine the longitudinal associations between latent classes of substance use and anxiety and depression scores among youth who use substances. This study uses data from three waves (Wave 1: 2017/18, Wave 2: 2018/19, and Wave 3: 2019/20) of the COMPASS study. Students in grades 9 and 10 who reported substance use at baseline (*n* = 738) report their substance use (alcohol, cannabis, cigarettes, and e-cigarettes) and anxiety and depression symptoms at each wave. A Repeated Measures Latent Class Analysis (RMLCA) is used to determine substance use classes, and mixed models are used to examine the associations between substance use classes and anxiety and depression. We identify three classes of substance use: (1) occasional alcohol and e-cigarette use, (2) escalating poly-substance use, and (3) consistent poly-substance use. After controlling for relevant covariates, consistent poly-substance use is associated with depression (Female OR: 1.24 [95%CI: 0.46, 2.02]; Male OR 1.13 [95%CI: 0.38, 1.87]) but not anxiety. Escalating poly-substance use is associated with depression among males (OR 0.72 [95%CI: 0.10, 1.33]). These findings should be taken into consideration when creating prevention programming and treatment strategies for adolescents. Substance use programming should be comprehensive, consider multiple substances, and be cognizant of symptoms of mental illness, particularly depression.

## 1. Introduction

Risk factors and negative outcomes associated with substance use have been well studied for individual substances in isolation (i.e., alcohol, cigarettes, cannabis) [1,2,3]. However, recent evidence indicates that 23% of Canadian students in grades 9–12 engage in poly-substance use, defined as the concurrent use of more than one substance within a specified period [4]. Prior cross-sectional research has consistently identified common patterns of substance use among adolescents, including a low use or no use group comprising the majority of adolescents, a single or dual substance use group, a moderate poly-substance use group, and finally a higher poly-substance use group [5]. Longitudinal research indicates that adolescents typically maintain substance use patterns over time but if they make a change, adolescents are more likely to increase than decrease the number of substances they use over time [6,7,8,9]. No studies to date have examined the role of recent increases in e-cigarette use among adolescents [10] in poly-substance use over time. This knowledge is vital in order to better understand the reality of youth substance use and to plan appropriate interventions and prevention programming.

Poly-substance use has also been linked with risk behaviours other than substance use (e.g., risky sexual behaviour, gambling) and negative health outcomes above and beyond using each substance in isolation, including poor mental health and mental ill-health [11,12,13,14,15,16,17,18,19,20]. Of note, poly-substance use has been associated with elevated symptoms of depression and anxiety among adolescents aged 11 to 18 in cross-sectional research [5,21,22,23,24], although previous longitudinal research has identified mixed results. A large US study found no associations between poly-substance use and depressive symptoms after controlling for other variables [9]. However, another study found that the onset of poly-substance use may worsen anxiety and depression symptoms among secondary school students who use substances [25], and yet another identified a transactional relationship between poly-substance use and depression symptoms, suggesting a more complex relationship between these two variables [13]. Adolescence is a critical juncture for addressing poor mental health and mental ill-health, as the majority of mental illnesses arise during adolescence and young adulthood [26]. Mental illnesses are a leading cause of disability in Canada and also present a significant current and predicted economic burden [27,28,29]. Early prevention and interventions may help maximize benefits to improve population mental health [30,31,32].

Previous longitudinal research has often examined poly-substance simply by counting the total substances used rather than by investigating specific substances used [13,25]. Only two studies to date have considered classes of substance use over time and their relationship with symptoms of mental illness [9,33]. For example, Brooks–Russell and colleagues used a Repeated Measures Latent Class Analysis (RMLCA) to examine substance use classes across grades 10 to 12 and their associations with depression symptoms [9]. Additionally, previous longitudinal studies have not explored sex differences, despite differences being identified in cross-sectional research [5,22]. Patterns of poly-substance use, anxiety, and depression are known to differ between female and male adolescents [5,22,34]. Female students report higher anxiety and depression symptoms than male students [34,35,36,37], and these sex differences tend to increase across adolescence [38,39]. Conversely, male students typically report higher levels of poly-substance use, both more substances and higher frequencies of use [5,16,22,40,41,42]. Having a better understanding of these relationships, including any sex-based differences, can help in tailoring prevention programming to the potential uniqueness of different groups.

Our research addresses these gaps in the existing literature by identifying patterns of substance use over time and their associations with anxiety and depression symptoms among male and female students. The study objectives are to, first, examine the longitudinal latent classes of substance use using RMLCA and, second, to examine their associations with anxiety and depression scores among youth who used substances in three waves of the COMPASS study (Wave 1: 2017/18, Wave 2: 2018/19, and Wave 3: 2019/20).

## 2. Materials and Methods

### 2.1. Design

The COMPASS study is a prospective cohort study that collects data annually from a convenience sample of students in British Columbia, Alberta, Ontario, and Quebec in grades 9 to 12 (Secondary I–V in Quebec). The current study used three waves of data from the COMPASS study. All procedures were approved by the University of Waterloo Office of Research Ethics (reference number 30118) and appropriate school board committees. A more detailed description of the COMPASS study is available online (https://uwaterloo.ca/compass-system/ accessed on 1 August 2021) or in print [43].

### 2.2. Participants

Overall, 122 schools (British Columbia, *n* = 16; Alberta, *n* = 8; Ontario, *n* = 61; and Quebec, *n* = 37) participated in the COMPASS study at Wave 1. Due to school closures because of COVID-19 in March 2020, only 29 schools (British Columbia, *n* = 5; Alberta, *n* = 5; Ontario, *n* = 17; and Quebec, *n* = 2) consistently completed the same questionnaire in all 3 waves and were included in this study. In these schools, 7084 students in grades 9 and 10 (Secondary III in Quebec, grade 9 equivalent) participated at Wave 1. Using an anonymous self-generated identification code, 2904 (41%) students were successfully linked across all three waves in the 29 schools [44]. Among the linked sample, there were a higher proportion of students who were in grade 9 and female and a lower proportion who reported substance use (Supplementary Materials Table S1). After missing data were removed, 1852 (64%) had complete data for all three waves. There were no significant differences between students with complete data and students who were removed due to incomplete data (Supplementary Materials Table S2). Finally, students who did not report any substance use at baseline (*n* = 1114, 60%) were removed for a final sample of 738 students.

### 2.3. Measures

#### 2.3.1. Substance Use

Substance use measures were consistent with national surveillance measures [45]. At each wave, students were asked to report *alcohol use* (“In the last 12 months, how often did you have a drink of alcohol that was more than just a sip?”), *cannabis use* (“In the last 12 months, how often did you use marijuana or cannabis? (A joint, pot, weed, hash)”), *cigarette use* (“Have you ever tried cigarette smoking, even just a few puffs?” and “On how many of the last 30 days did you smoke one or more cigarettes?”), and *e-cigarette use* (“Have you ever tried an electronic cigarette, also known as an e-cigarette?” and “On how many of the last 30 days did you use an e-cigarette?”). Each substance was categorized into one of four categories: no use, ever/less than monthly use, monthly use, or weekly use. Students who had missing data for all four substances were removed from the analyses.

#### 2.3.2. Anxiety

The Generalized Anxiety Disorder 7 (GAD-7) scale [46] was used to assess generalized anxiety symptoms at each wave of the study. The GAD-7 reports on self-perceived feelings of worry, fear, and irritability over a 2-week period. Students were asked how often they were bothered by each symptom with the response options: “Not at all”, “Several days”, “Over half the days”, or “Nearly every day”. Responses were scored from 0 to 3, respectively, and summed. Total scores range from 0 to 21 and higher total scores indicate greater anxiety symptoms. The GAD-7 had an alpha coefficient of 0.91 for females and 0.89 for males at Wave 1. As anxiety scores were used as both independent and dependent variables, students with missing scores were removed from the sample.

#### 2.3.3. Depression

The Centre for Epidemiological Studies Depression Scale (CES-D-10) [47,48] was used to assess depression symptoms at each wave of the study. Items assess characteristics of clinical depression, including negative affect, anhedonia, and somatic symptoms, such as, “I felt everything I did was an effort” and “I could not get ‘going.’” Students were asked how often they experienced each symptom within the last 7 days, with the response options: “None or less than 1 day”, “1–2 days”, “3–4 days”, or “5–7 days”. Responses were scored from 0 to 3, respectively, and summed. Total scores range from 0 to 30 and higher total scores indicate greater depressive symptoms. The CES-D-10 had an alpha coefficient of 0.70 for females and 0.75 for males at Wave 1. As depression scores were used as both independent and dependent variables, students with missing scores were removed from the sample.

#### 2.3.4. Covariates

Poly-substance use, anxiety, and depression are associated with other risky behaviour as well as family and friend support [12,16,17,18,19,20,49,50,51,52]. Skipping school was used as a measure of risky student behaviour. Students were asked, “In the last 4 weeks, how many classes did you skip when you were not supposed to?” Students who reported any number of classes skipped were categorized as truant. To ascertain whether students felt they had family or friend support, they were asked how much they agreed with the statements, “I can talk about my problems with my family/friends.” Students who selected “Agree” or “Strongly Agree” were categorized as having family or friend support. These variables were assessed at Wave 1.

Consistent with other Canadian adolescent health research [45], sex (male, female), grade (9, 10), ethnicity (white, non-white), and weekly spending money (zero, CAD 1–20, 21–100, 100+, don’t know/missing), were assessed at Wave 1 and included as demographic covariates.

### 2.4. Analyses

To create substance use classes and examine their associations with anxiety and depression, a Repeated Measures Latent Class Analysis (RMLCA) [53] was implemented using Mplus 8.2 (Muthen & Muthen, Los Angeles, CA, USA). RMLCA is an application of the Latent Class Analysis (LCA) that identifies latent classes over time [53]. First, a series of RMLCA models were fit to determine the number of classes to best fit the data. Categorical indicators of alcohol use, cannabis use, cigarette use, and e-cigarette use were used as latent class indicators. To establish the best fitting solution, we started with a 1-class solution and added classes until good fit was no longer obtained. We used log-likelihood, AIC, BIC, and the Lo–Mendell–Rubin adjusted likelihood ratio test (LMRT) as indicators of model fit. Lower log-likelihood, AIC, and BIC values indicate better model fit [54]. The LMRT tests whether a model with *k* classes fits better than a model with *k* − 1 classes where a significant result indicates better fit [55]. These model selection criteria, combined with model interpretability, were used to place participants into the appropriate latent classes. While entropy was not used for model selection, it is reported as an indicator of classification from 0 to 1 with larger values indicating better latent class separation [53,56]. The TYPE = COMPLEX and CLUSTER commands were used to account for the nesting of students within schools. Based on the previous evidence of sex differences, separate models were conducted for females and males [5,22]. Once the most parsimonious solution was determined, students were assigned to a latent class using most likely class membership [57].

Descriptive statistics were used to examine the Wave 1 characteristics of the linked longitudinal sample by substance use class and the prevalence of number of substances used, poly-substance use, anxiety, and depression in each wave of the study.

After the RMLCA was completed, we used the PROC MIXED function in SAS (SAS Institute Inc., Cary, NC, USA) to fit linear mixed effects regression models. Using 3 years of data, the models tested the effects of engaging in the different classes of substance use on adolescents’ anxiety and depression trajectories over time. All mixed effects models included a random intercept term to account for the within-student correlation of response over time as well as students nesting within schools. For models where a significant main effect was seen, an interaction with the wave was tested, however, no significant effects were found. Two sets of models were run, the first controlled for grade, ethnicity, weekly spending money, friend support, family support, and skipping school. The second set of models controlled for depression in the anxiety models and vice versa.

## 3. Results

Approximately half of the sample was female (53%) and in grade 9 (46%). At Wave 1, the sample had a mean anxiety score of 6.8 (SD 5.8), and a mean depression score of 9.2 (SD 6.2).

### 3.1. Repeated Measures Latent Class Analysis

**Figure 1:**
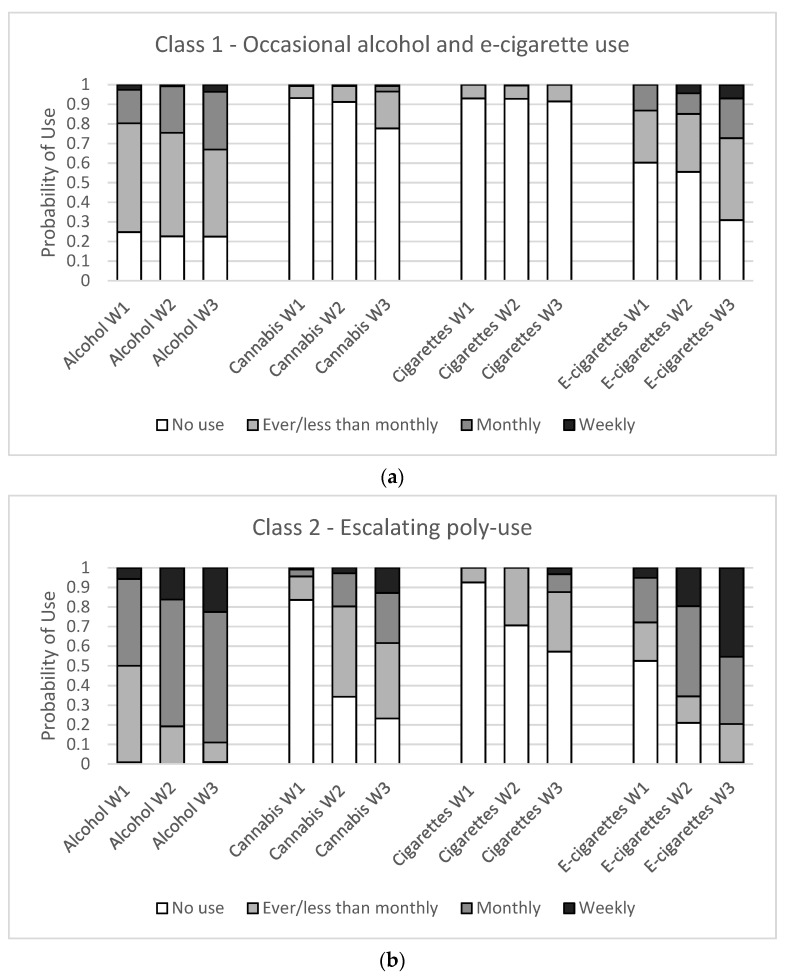

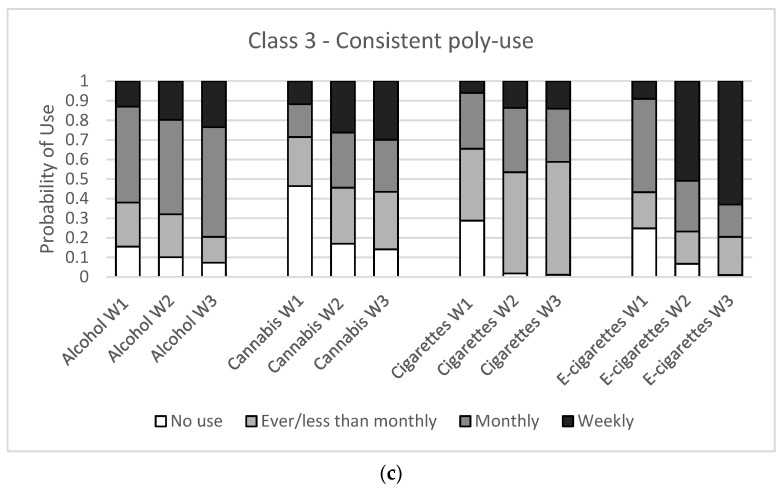
Substance use item probabilities for a three-class latent class model for females in three waves of the COMPASS study (Wave 1: 2017/18, Wave 2: 2018/19, Wave 3: 2019/20) in British Columbia, Alberta, Ontario, and Quebec, Canada. (**a**) Class 1—occasional alcohol and e-cigarette use, (**b**) Class 2—escalating poly-use (**c**) Class 3—consistent poly-use.

**Figure 2:**
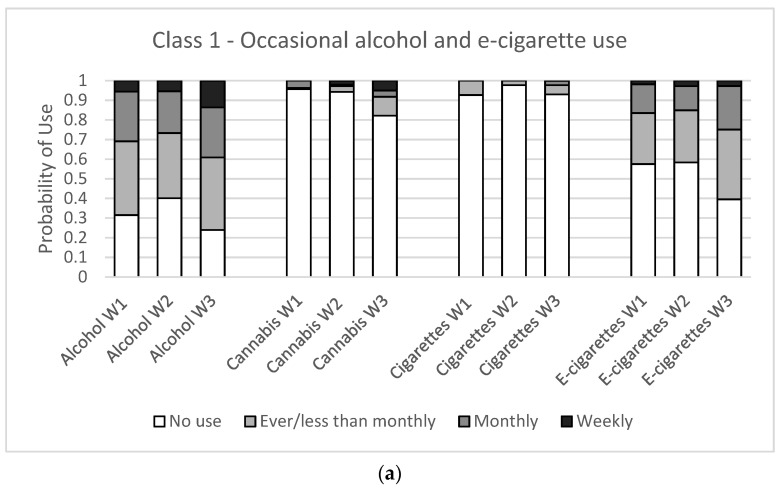

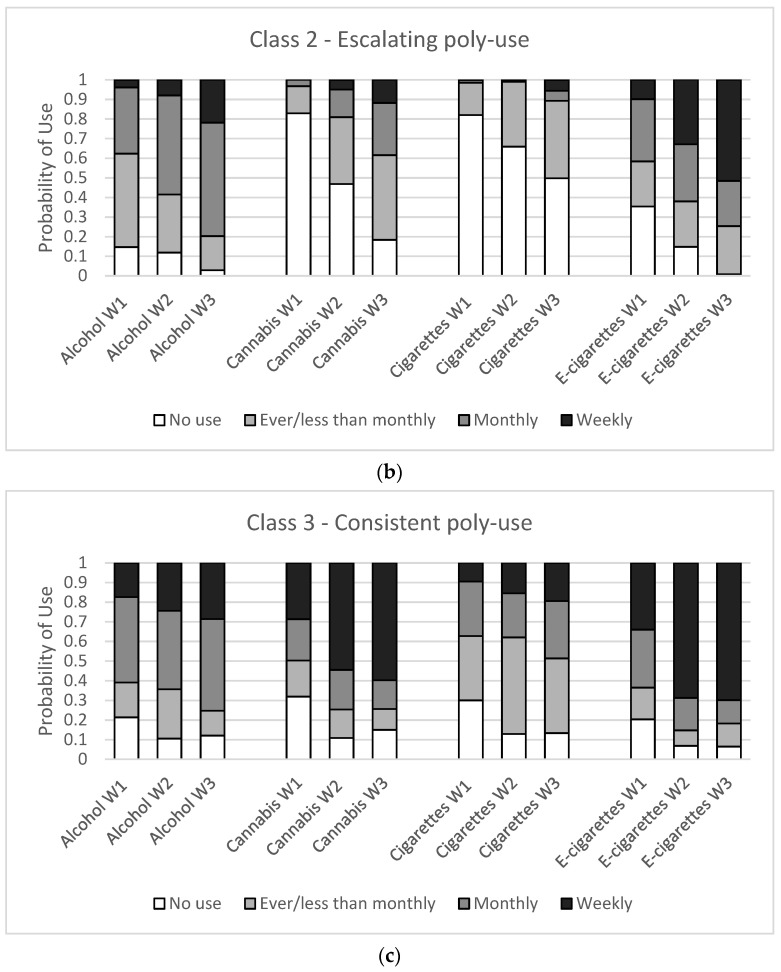
Substance use item probabilities for a four-class latent class model for males in three waves of the COMPASS study (Wave 1: 2017/18, Wave 2: 2018/19, Wave 3: 2019/20) in British Columbia, Alberta, Ontario, and Quebec, Canada. (**a**) Class 1—experimental alcohol and e-cigarette use, (**b**) Class 2—escalating poly-use (**c**) Class 3—consistent poly-use.

To determine the best model, we examined model fit statistics for one to six latent classes (Table 1). A three-class model was selected as the best fitting model as it had lower values for the model selection criteria and the best interpretability. The three classes identified in this study were (1) occasional alcohol and e-cigarette use (*occasional*), (2) escalating poly-substance use (*escalating*), and (3) consistent poly-substance use (*consistent*). While classes were similar among females and males, they had slightly different interpretations and are described in Table 2 and Figure 1 and Figure 2.

Among female students (Figure 1), the occasional class (45%) was characterized by consistent “less than” monthly alcohol use and increasing “ever” e-cigarette use across waves. The escalating class (29%) was characterized by increasing monthly alcohol use, increasing “less than” monthly cannabis use, and increasing monthly and weekly e-cigarette use. Students in the escalating class were primarily using alcohol and e-cigarettes at Wave 1 and by Wave 3 were engaging in some cannabis and cigarette use. The consistent class (26%) was characterized by regular monthly alcohol use, increasing “ever” and monthly cannabis use, regular “ever” and monthly cigarette use, and increasing monthly and weekly e-cigarette use. Students in the consistent class were engaging with all four substances at Wave 1 and this remained consistent over time.

Among male students (Figure 2), the occasional class (42%) was characterized by consistent “less than” monthly alcohol use and “ever” e-cigarette use. The escalating class (35%) was characterized by increasing monthly alcohol use, increasing “less than” monthly cannabis use, and increasing monthly and weekly e-cigarette use. Students in the escalating class were primarily using alcohol and e-cigarettes at Wave 1 and by Wave 3 were engaging in some cannabis and cigarette use. Finally, the consistent class (23%) was characterized by consistent monthly alcohol use, increasing weekly cannabis use, consistent “ever” and monthly cigarette use, and increasing weekly e-cigarette use. Students in the consistent class were engaging with all four substances at Wave 1 and this remained consistent over time.

### 3.2. Regression Results

Regression coefficients for all models are found in Table 3. Among female students, results from the Anxiety Model 1 suggested that the consistent class had significantly higher anxiety scores than the occasional class. However, after controlling for the depression score, this relationship was no longer significant. Results from the Depression Model 1 indicated that the consistent class had significantly higher anxiety scores than the occasional class; this relationship held after controlling for anxiety in Model 2 (β = 1.24; 95% CI: 0.46, 2.02).

Among male students, the escalating class and consistent class had significantly higher anxiety scores than the occasional alcohol and e-cigarette use class in the Anxiety Model 1. However, this relationship was no longer significant after controlling for depression in the Anxiety Model 2. The escalating class and consistent class had significantly higher depression scores than the occasional class in both the Depression Model 1 and Depression Model 2. This result indicates that after controlling for all other variables, those in the escalating class had 0.72 higher depression scores (95% CI: 0.10, 1.33) and those in the consistent class had 1.13 higher depression scores (95% CI: 0.38, 1.87) than those in the occasional class.

## 4. Discussion

The purpose of this study was to examine how latent classes of substance use were associated with anxiety and depression scores over time among adolescents who used substances. We identified three unique classes of substance use: occasional alcohol and e-cigarette use, escalating poly-substance use, and consistent poly-substance use. The occasional group was the largest class representing just under half of our sample, whereas the escalating and consistent groups were roughly the same size each, at around a quarter of our sample. Notably, there were no significant interactions between substance use classes and time indicating that any differences in depression and anxiety scores were consistent across time. After controlling for depression, substance use classes were not associated with anxiety among female or male students. In contrast, after controlling for anxiety, the escalating poly-use class among males and the consistent poly-use class among both females and males were associated with higher depression scores than the occasional class. As such, our data suggest that while poly-substance use may have a role in the worsening of depression symptoms, depression plays a larger role in the worsening of anxiety symptoms than poly-substance use, although additional investigation is required to strengthen the evidence on the temporality of these associations.

The longitudinal classes of substance use identified in this study differed from existing research; however, there were significant differences in samples and methodology that could explain these differences. For example, in the US, Brooks–Russell et al. identified a four-class model that included (1) alcohol and other drug use, (2) tobacco, alcohol, and other drug use, (3) increasing multiple substance use, and (4) decreasing multiple substance use [9]. There are some similarities with this work including identifying students who maintained their substance use over time and those who increased their substance use over time. However, we did not include as many substances (i.e., ecstasy, amphetamines, cocaine) in our analyses. Additionally, we did not identify a decreasing use group over time. While some students in our study did decrease their use over time (17%), it was not a common enough pattern to be identified as a unique class. McKelvey et al. also used a similar approach; their LCA results identified two distinct classes of substance use: (1) a limited range group that reported use of tobacco, alcohol, and cannabis and (2) an extended range group that also reported use of additional substances (i.e., cocaine, ecstasy, misused prescriptions) [33]. However, their sample comprised only smokers, so results are not necessarily comparable, as only 4% of adolescents in the current study reported current smoking. Notably, both of these studies are older and did not examine the recent increase in e-cigarette use among adolescents [10]. Our results indicate that e-cigarette use was a consistent component of each substance use class and therefore is important to include in future work examining poly-substance use over time.

Next, we identified a notable difference between female and male substance use classes. Male students in the consistent poly-use class had a higher probability of weekly cannabis and e-cigarette use. Over half of males in this class reported weekly cannabis use and over two thirds reported weekly e-cigarette use by Wave 3, whereas these numbers were approximately one-third and one-half, respectively, among female students. The high prevalence of the weekly use of both substances warrants further investigation into whether they are being used simultaneously and the preferred mode of cannabis use in this group. As these students are regularly using e-cigarettes, they may also be consuming cannabis by vaping.

In regression analyses, we found that after controlling for depression symptoms, consistent poly-substance use was no longer associated with anxiety symptoms. This is in contrast to previous findings where poly-substance use was associated with elevated anxiety symptoms compared to single substance use [25]. Instead, we found that over time, anxiety symptoms were primarily explained by depression symptoms. Specifically, we identified that a 10-unit increase in depression symptoms (indicative of clinically relevant depression symptoms) was associated with a 6.3-unit increase in anxiety score among females and a 6.4-unit increase in anxiety score among males [47]. This observed comorbidity was expected, as it is common to have overlapping symptoms [22]. In a previous examination of the baseline sample of this study, 51% of those who reported clinically relevant symptoms of anxiety or depression, reported clinically relevant symptoms of both and few reported symptoms of anxiety alone (11%) [22].

Finally, we found that consistent poly-substance use was associated with depressive symptoms even after controlling for anxiety symptoms. This is consistent with the majority of previous literature [13,25,33]; however, one study found no association after adjusting for peer substance use [9]. This finding is consistent with the opponent process model of addiction which hypothesizes that substance use in adolescence predicts future depressive symptoms [58]. It has been proposed that substance use promotes an immediate mood boost (“appetitive process”) followed by a slow decrease in mood (“opponent process”), which over time becomes stronger than the “appetitive process” and negatively affects mood over time. However, it is important to note that we did not see significant interactions with time, meaning that baseline differences between groups held over time. This means that increased substance use over time did not result in increasingly worse depression symptoms compared to the lowest use class. Additionally, the increasing poly-substance use class was associated with depression symptoms among male students only. This was surprising, as female students began with worse anxiety and depression scores, perhaps indicating a ceiling effect of anxiety and depression symptoms among female students.

### Strengths and Limitations

This study made use of a longitudinal sample. The COMPASS study also uses an active-information passive-consent protocol to encourage participation and honest reporting, which has been shown to be particularly important in substance use and mental health research [43,59,60,61]. Finally, we made use of validated scales to measure anxiety and depression symptoms [47,62].

However, this study was not without limitations. First, the COMPASS survey measured concurrent (i.e., multiple substances being used over the same time period such as the past 30 days or past 12 months), not simultaneous (i.e., multiple substances being used in the same instance) poly-substance use and therefore, we cannot draw any conclusions about simultaneous substance use. Second, the COMPASS study is not representative of all Canadian secondary school students, limiting the generalizability of results. Third, there are several limitations with the questionnaire that could result in participants under-reporting their substance use. These include the illicit nature of the substances for underage youth, a limited number of substances included on the questionnaire, and in Waves 1 and 2, the lack of a definition of an e-cigarette or listing of brands on the questionnaire. Fourth, we were lacking measures of peer or family substance use which have been associated with early initiation and escalating use through adolescence [63]. However, this study made use of variables indicating family and friends support which have been positively and negatively associated with poly-substance use, respectively [16]. Additionally, there were no measures available of parental psychopathology, which is a significant risk factor for children [64,65]. Finally, only two-fifths of participants were linked over time. This is consistent with previous research findings where older, male, and youth who use substances are less likely to be linked over time [44,66]. We also identified that those with higher anxiety and depression scores were less likely to be linked over time. This may have resulted in an underestimation of substance use rates and their associations with anxiety and depression scores.

## 5. Conclusions

This research gives novel insights into how Canadian adolescents use substances over time. Three classes of substance use emerged among those who used substances: (1) occasional alcohol and e-cigarette use, (2) escalating poly-substance use, and (3) consistent poly-substance use. We did not identify significant interactions between time and group membership; however, we did identify a link between poly-substance use and depression. Over time, consistent poly-substance use was associated with depression but not anxiety, and escalating poly-substance use was associated with depression among males. These findings should be taken into consideration when creating prevention programming and treatment strategies for adolescents. Substance use programming should be comprehensive, consider multiple substances, and be cognizant of symptoms of mental illness, particularly depression. Future research should continue to include e-cigarette use and consider modes of cannabis use when examining poly-substance use.

## Figures and Tables

**Table 1:** Model fit indices for one through six latent class models of substance use among female and male substance users in three waves of the COMPASS study (Wave 1: 2017/18, Wave 2: 2018/19, Wave 3: 2019/20) in British Columbia, Alberta, Ontario, and Quebec, Canada.

Number of Classes	Log-Likelihood	FP	AIC	BIC	LMRT *p*-Value	Entropy
**Female (*n* = 388)**						
1	−5194.0	36	10,459.9	10,602.5	-	1.00
2	−4665.3	73	9476.7	9765.8	0.00	0.90
3	−4535.5	110	9291.0	9726.7	0.80	0.86
4	−4432.2	147	9158.4	9740.7	0.77	0.88
5	−4371.6	184	9111.3	9840.1	0.78	0.89
6	−4317.8	221	9077.6	9953.0	0.78	0.90
**Male (*n* = 350)**						
1	−4847.1	36	9766.3	9905.2	-	1.00
2	−4400.3	73	8946.6	9228.2	0.07	0.87
3	−4287.1	110	8794.3	9218.6	0.77	0.86
4	−4187.2	147	8668.4	9235.6	0.60	0.88
5	−4103.8	184	8575.7	9285.5	0.76	0.91
6	−4055.1	221	8552.2	9404.8	0.77	0.93

FP = Free Parameters; AIC = Akaike information criterion; BIC = Bayesian information criterion; LMRT = Lo–Mendell–Rubin Test.

**Table 2:** Wave 1 characteristics of the linked longitudinal sample of adolescents participating in three waves of the COMPASS study (Wave 1: 2017/18, Wave 2: 2018/19, Wave 3: 2019/20) in British Columbia, Alberta, Ontario, and Quebec, Canada. (*n* = 738).

Wave 1 Variables	Female (*n* = 388)						Male (*n* = 350)					
	Class 1 (*n* = 175)		Class 2 (*n* = 113)		Class 3 (*n* = 100)		Class 1 (*n* = 146)		Class 2 (*n* = 124)		Class 3 (*n* = 80)	
	*n*	%	*n*	%	*n*	%	*n*	%	*n*	%	*n*	%
**Grade**												
9	87	49.7	54	47.8	43	43.0	73	50.0	55	44.4	26	32.5
10	88	50.3	59	52.2	57	57.0	73	50.0	69	55.6	54	67.5
**Ethnicity**												
White	118	67.4	93	82.3	79	79.0	100	68.5	98	79.0	48	60.0
Non-White	57	32.6	20	17.7	21	21.0	46	31.5	26	21.0	32	40.0
**Weekly spending money**												
Zero	32	18.3	14	12.4	15	15.0	32	21.9	17	13.7	11	13.8
CAD 1–20	69	39.4	38	33.6	25	25.0	45	30.8	39	31.5	19	23.8
CAD 21–100	32	18.3	26	23.0	24	24.0	40	27.4	34	27.4	22	27.5
CAD 100+	17	9.7	17	15.0	22	22.0	12	8.2	18	14.5	19	23.8
Don’t know/missing	25	14.3	18	15.9	14	14.0	17	11.6	16	12.9	9	11.3
**Anxiety score** **(GAD-7; mean, SD)**	8.3	5.9	7.2	5.2	10.5	6.3	4.3	4.5	5.0	5.0	6.2	5.4
**Depression score** **(CESD; mean, SD)**	9.9	6.7	9.1	5.0	13.7	7.2	6.6	4.5	7.6	5.5	9.2	5.8

Note: Class 1 = occasional alcohol and e-cigarette use, Class 2 = escalating poly-substance use, and Class 3 = consistent poly-substance use.

**Table 3:** Mixed effects regression coefficients between substance use class and anxiety and depression scores over time among of adolescents participating in three waves of the COMPASS study (Wave 1: 2017/18, Wave 2: 2018/19, Wave 3: 2019/20) in British Columbia, Alberta, Ontario, and Quebec, Canada. (*n* = 738).

Variables	Anxiety Model 1 β (95% CI)	Anxiety Model 2 β (95% CI)	Depression Model 1 β (95% CI)	Depression Model 2 β (95% CI)
**Female**				
**Year**				
Wave 1	0.00	0.00	0.00	0.00
Wave 2	**0.65 (0.11, 1.20)**	0.04 (−0.40, 0.48)	**0.98 (0.39, 1.57)**	**0.50 (0.02, 0.97)**
Wave 3	**0.98 (0.43, 1.53)**	0.41 (−0.03, 0.86)	**0.90 (0.31, 1.49)**	0.18 (−0.30, 0.65)
**Substance Use Class**				
Occasional alcohol and e-cigarette use	0.00	0.00	0.00	0.00
Escalating poly-use	−0.45 (−1.53, 0.63)	−0.10 (−0.78, 0.59)	−0.56 (−1.72, 0.60)	−0.23 (−0.97, 0.51)
Consistent poly-use	**2.07 (0.94, 3.20)**	0.33 (−0.39, 1.06)	**2.76 (1.54, 3.98)**	**1.24 (0.46, 2.02)**
**Depression score**	**-**	**0.63 (0.59, 0.67)**	**-**	**-**
**Anxiety Score**	**-**	**-**	**-**	**0.74 (0.69, 0.78)**
**Male**				
**Year**				
Wave 1	0.00	0.00	0.00	0.00
Wave 2	0.15 (−0.39, 0.69)	−0.09 (−0.55, 0.37)	0.38 (−0.19, 0.95)	0.27 (−0.22, 0.76)
Wave 3	**0.57 (0.03, 1.11)**	−0.09 (−0.56, 0.37)	**1.04 (0.47, 1.61)**	**0.64 (0.15, 1.12)**
**Substance Use Class**				
Occasional alcohol and e-cigarette use	0.00	0.00	0.00	0.00
Escalating poly-use	**1.00 (0.02, 1.98)**	0.09 (−0.50, 0.69)	**1.42 (0.41, 2.43)**	**0.72 (0.10, 1.33)**
Consistent poly-use	**1.28 (0.10, 2.47)**	−0.02 (−0.74, 0.71)	**2.03 (0.80, 3.26)**	**1.13 (0.38, 1.87)**
**Depression score**	**-**	**0.64 (0.60, 0.68)**	-	-
**Anxiety Score**	-	-	-	**0.70 (0.66, 0.74)**

Anxiety Model 1 and Depression Model 1 also controlled for grade, ethnicity, weekly spending money, friend support, family support, and skipping school. Anxiety Model 2 additionally controlled for depression score. Depression Model 2 additionally controlled for anxiety score. Bold indicates significance <0.05.

## Data Availability

The datasets generated and analysed for this study will not currently be shared as this is an on-going study; however, access to the data supporting the findings of this study can be requested at https://uwaterloo.ca/compass-system/information-researchers (accessed on 1 August 2021).

## Supplementary Materials

The following are available online at https://www.mdpi.com/article/10.3390/ijerph181910468/s1, Table S1: Comparison of 3-year (Wave 1: 2017/18 to Wave 3: 2019/20) unlinked (ref) vs. linked students at Wave 1 in the COMPASS study, Table S2: Comparison of Wave 1 characteristics of students with complete data versus students who were removed due to incomplete data in a 3-year (Wave 1: 2017/18 to Wave 3: 2019/20) linked sample of grade 9 and 10 students in the COMAPSS study.
