# Supplementary material for: Associations between Longitudinal Patterns of Substance Use and Anxiety and Depression Symptoms among a Sample of Canadian Secondary School Students
Source: Int J Environ Res Public Health. 2021 Oct 5;18(19):10468. doi: 10.3390/ijerph181910468 (PMC8507734; doi:10.3390/ijerph181910468)
Supplement: Supplementary file 1 [file ijerph-18-10468-s001.zip › ijerph-1355130-supplementary.pdf]

Supplementary Materials

Table S1: Comparison of 3-year (Wave 1: 2017/18 to Wave 3: 2019/20) unlinked (ref) vs linked students at Wave 1 in the COMPASS study.

**Wave 1 variables**	**Unlinked (n=4180)**		**Linked (n=2904)**		**Chi-square or t-test p-value**	**Cramer's V**
	**n**	**%**	**n**	**%**		
**Grade**						
9	1903	45.53	1597	54.99	<.0001	-0.0931
10	2277	54.47	1307	45.01		
**Sex**						
Female	1872	45.26	1582	54.59	<.0001	-0.0918
Male	2264	54.74	1316	45.41		
Missing	44		6			
**Ethnicity**						
White	2452	59.14	1933	66.77	<.0001	-0.0774
Non-White	1694	40.86	962	33.23		
Missing	34		9			
**Weekly spending money**						
Zero	799	19.11	651	22.42	<.0001	0.0817
\$1-\$20	1335	31.94	1025	35.30		
\$21-\$100	928	22.20	527	18.15		
\$100+	463	11.08	228	7.85		
Don't know/missing	655	15.67	473	16.29		
**Past 12-month alcohol use**						
No use	2299	56.38	1969	68.32	<.0001	0.1475
Ever/less than monthly	657	16.11	456	15.82		
Monthly	811	19.89	370	12.84		
Weekly	311	7.63	87	3.02		
Missing	102		22			
**Past 12-month cannabis use**						
No use	3178	77.97	2602	90.28	<.0001	0.1763
Ever/less than monthly	276	6.77	131	4.55		
Monthly	209	5.13	83	2.88		
Weekly	413	10.13	66	2.29		
Missing	104		22			

**Ever/Past 30-day cigarette use**						
No use	3097	75.02	2588	89.33	<.0001	0.1899
Ever/less than monthly	526	12.74	198	6.83		
Monthly	240	5.81	82	2.83		
Weekly	265	6.42	29	1.00		
Missing	52		7			
**Ever/Past 30-day e-cigarette use**						
No use	2623	64.30	2225	77.61	<.0001	0.1523
Ever/less than monthly	537	13.16	262	9.14		
Monthly	578	14.17	288	10.05		
Weekly	341	8.36	92	3.21		
Missing	101		37			
**Anxiety score (GAD-7; mean, SD)**	6.56	6.09	6.02	5.49	0.0002	
Missing	390		208			
**Depression score (CESD; mean, SD)**	9.27	6.22	8.30	5.70	<.0001	
Missing	801		448			

Note: Missing values without corresponding percentages were not included in chi-square tests.

Table S2: Comparison of Wave 1 characteristics of students with complete data versus students who were removed due to incomplete data in a 3-year (Wave 1: 2017/18 to Wave 3: 2019/20) linked sample of grade 9 and 10 students in the COMAPSS study.

	**Incomplete (n=1052)**		**Complete (n=1852)**		**Chi-square or t-test p-value**	**Cramer's V**
**Wave 1 variables**	**n**	**%**	**n**	**%**		
**Grade**						
9	599	56.94	998	53.89	0.1121	0.0295
10	453	43.06	854	46.11		
**Sex**						
Female	565	54.02	1017	54.91	0.6409	-0.0087
Male	481	45.98	835	45.09		
Missing	6					
**Ethnicity**						
White	704	67.50	1229	66.36	0.5330	0.0116
Non-White	339	32.50	623	33.64		
Missing	9					
**Weekly spending money**						
Zero	238	22.62	413	22.30	0.1241	0.0499
$1-$20	395	37.55	630	34.02		
$21-$100	169	16.06	358	19.33		
$100+	76	7.22	152	8.21		
Don't know/missing	174	16.54	299	16.14		
**Past 30-day alcohol use**						
No use	716	69.05	1253	67.91	0.5702	0.0264
Ever/less than monthly	157	15.14	299	16.21		
Monthly	128	12.34	242	13.12		
Weekly	36	3.47	51	2.76		
Missing	15		7			
**Past 30-day cannabis use**						
No use	920	89.23	1682	90.87	0.3936	0.0322
Ever/less than monthly	48	4.66	83	4.48		
Monthly	35	3.39	48	2.59		
Weekly	28	2.72	38	2.05		
Missing	21		1			
**Past 30-day cigarette use**						
No use	920	88.04	1668	90.06	0.1131	0.0454

Ever/less than monthly	82	7.85	116	6.26		
Monthly	28	2.68	54	2.92		
Weekly	15	1.44	14	0.76		
Missing	7					
**Past 30-day e-cigarette use**						
No use	786	76.53	1439	78.21	0.7814	0.0194
Ever/less than monthly	99	9.64	163	8.86		
Monthly	108	10.52	180	9.78		
Weekly	34	3.31	58	3.15		
Missing	25		12			
**Anxiety score (GAD-7; mean, SD)**	5.93	5.49	6.06	5.48	0.5622	
Missing	208					
**Depression score (CESD; mean, SD)**	8.64	5.83	8.18	5.65	0.0860	
Missing	448					
